# Control Costs, Enhance Quality, and Increase Revenue in Three Top General Public Hospitals in Beijing, China

**DOI:** 10.1371/journal.pone.0072166

**Published:** 2013-08-16

**Authors:** Lue-Ping Zhao, Guo-Pei Yu, Hui Liu, Xie-Min Ma, Jing Wang, Gui-Lan Kong, Yi Li, Wen Ma, Yong Cui, Beibei Xu, Na Yu, Xiao-Yuan Bao, Yu Guo, Fei Wang, Jun Zhang, Yan Li, Xue-Qin Xie, Bao-Guo Jiang, Yang Ke

**Affiliations:** 1 Peking University Medical Informatics Center, Peking University, Beijing, China; 2 Fred Hutchinson Cancer Research Center, Seattle, Washington, United States of America; 3 New York Medical College, Valhalla, New York, United States of America; 4 School of Public Health, Peking University Health Science Center, Peking University, Beijing, China; 5 Department of Hospital Management of Peking University Health Science Center, Neurology of Department of Peking University Third Hospital, Peking University, Beijing, China; 6 Department of Hospital Management of Peking University Health Science Center, Neurology Department of Peking University First Hospital, Peking University, Beijing, China; 7 Beijing Public Health Information Center, Beijing, China; 8 Peking University Health Science Center, Peking University, Beijing, China; Old Dominion University, United States of America

## Abstract

**Background:**

With market-oriented economic and health-care reform, public hospitals in China have received unprecedented pressures from governmental regulations, public opinions, and financial demands. To adapt the changing environment and keep pace of modernizing healthcare delivery system, public hospitals in China are expanding clinical services and improving delivery efficiency, while controlling costs. Recent experiences are valuable lessons for guiding future healthcare reform. Here we carefully study three teaching hospitals, to exemplify their experiences during this period.

**Methods:**

We performed a systematic analysis on hospitalization costs, health-care quality and delivery efficiencies from 2006 to 2010 in three teaching hospitals in Beijing, China. The analysis measured temporal changes of inpatient cost per stay (CPS), cost per day (CPD), inpatient mortality rate (IMR), and length of stay (LOS), using a generalized additive model.

**Findings:**

There were 651,559 hospitalizations during the period analyzed. Averaged CPS was stable over time, while averaged CPD steadily increased by 41.7% (P<0.001), from CNY 1,531 in 2006 to CNY 2,169 in 2010. The increasing CPD seemed synchronous with the steady rising of the national annual income per capita. Surgical cost was the main contributor to the temporal change of CPD, while medicine and examination costs tended to be stable over time. From 2006 and 2010, IMR decreased by 36%, while LOS reduced by 25%. Increasing hospitalizations with higher costs, along with an overall stable CPS, reduced IMR, and shorter LOS, appear to be the major characteristics of these three hospitals at present.

**Interpretations:**

These three teaching hospitals have gained some success in controlling costs, improving cares, adopting modern medical technologies, and increasing hospital revenues. Effective hospital governance and physicians' professional capacity plus government regulations and supervisions may have played a role. However, purely market-oriented health-care reform could also misguide future healthcare reform.

## Introduction

As the world is witnessing impressive economic development in China with the annual Gross Domestic Product (GDP) exceeding 10% per year for past decade [1], the international community has a keen eye on how Chinese government reforms its health-care system to provide care to approximately 1.4 billion people, a similar challenge faced by other developing and populous countries, like India. Recognizing this enormous challenge, Chinese government, in particular, Ministry of Health, has led multiple waves of health-care reforms, since 1992. In the earlier reforms, public hospitals were asked to follow the model of market economy, raising their own funds to maintain and improve their service deliveries to general public. While all public hospitals were given a considerable autonomy in their use of revenues, the governance structure is largely unchanged, despite tighten regulations. Due to financial pressure, public hospitals had to generate additional incomes through selling medicines. Inevitably, such a practice resulted in the increases of over-prescriptions and health-care costs, placing financial burden on both patients and government. In 2009, Chinese government launched another wave of health-care reform, aiming at health-care insurance coverage. Within three years, this reform covered over 95% of the entire population [2]. As expected, more people, especially those from rural areas, can now afford basic health care. Nevertheless, the surge of patient population put even more pressure on already straddled health-care resources.

In this ever-changing environment, top rank public hospitals (all hospitals are ranked as 1, 2, 3B and 3A progressively) are balancing multiple interests between their patients, physicians, hospitals, and government (acting as an insurance in some sense), with the hope of enhancing care quality, controlling costs, improving well-being of health-care providers, and increasing hospital revenues. How are these hospitals doing over the past few years? Many healthcare policy experts or analysts have written about successes and failures of the China health-care reform [3], [4]. In the broader internet community, there is also a wide range of discussions and debates.

Using empirical data taken directly from hospitals, our objective is to characterize inpatient care during the healthcare reform period, so as to complement expert opinions and non-hospital survey data. Towards this objective, we have chosen three teaching hospitals of Peking University, all of which are premier hospitals in China. While they are not representative of general hospitals in China, experiences of these three hospitals, just as a tip of iceberg, would offer insights into how hospitals are performing in light of ongoing healthcare reform. Using Hospitalization Summary Report (HSR) between 2006 and 2010, we examined temporal trends of cost per stay (CPS), cost per day (CPD), inpatient mortality rate (IMR), and length of stay (LOS) over this period. Basing on data from these three hospitals, we aim to characterize these three teaching hospitals in China, during this period.

## Methods

### Data Source

All hospitals in Beijing are regulated by the Beijing Municipal Health Bureau, Beijing, China. They are required to submit every HSR to a centralized health information system, in accordance with the administrative requirement of the Ministry of Health (full document is posted on the official website: http://www.bjhb.gov.cn/zwfwq/ztlm/drg/zidianku/201110/t20111008_41261.htm).

The HSR includes information on basic demographics, admission and discharge dates, eight hospitalization diagnostic names and corresponding ICD-10-CM codes (1 primary and 7 accompanied diagnoses), treatments, outcomes of hospitalization, and hospitalization costs. Electronic submission for HSRs has been implemented since 2004. The HSRs in three affiliated hospitals of Peking University were made available to us, with permission from the Beijing Municipal Health Bureau, and without personal identification information for protecting patient privacy. In addition, three hospitals were also labeled as A, B and C, without further disclosure. The Bureau has no influence on the design and conduct of this study.

### Statistical Analysis

Between 2006 and 2010, Chinese currency underwent a substantial inflation. Therefore, throughout the manuscript (unless otherwise noted), we adjusted for inflation by dividing a monetary time series by the Consumer Price Index (CPI) when hospitalization costs were calculated. This inflation-adjusted currency reflects the currency value in the end of 2010. Annual CPI data in China were obtained from the National Bureau of Statistics of China (http://www.stats.gov.cn/english/). Each cost was multiplied by the CPI from the relevant month to the end of year 2010.

We used generalized additive models (GAM) [5] to describe the temporal trends of CPS, CPD, IMR and LOS from year 2006 to 2010. In the model, the key outcome variables were regressed on the discharge dates without imposing any parametric assumption on the variable “time”. In addition, different link functions were used in the GAMs. The linear-link was used if outcome variables were continuous, while the logistic-link was used if they were binary. Naturally, we used GAMs to adjust for possible confounding variables, including hospital, age, gender, and disease severity at admission. To compute changing percentages of CPS, CPD, IMR and LOS between 2006 and 2010, we used the predicted values at the mid-year of 2006 and the mid-year of 2010 (marked on the figures), and took their percentages for reporting purpose in order to achieve consistency with CPI computation and also statistical stability. All statistical analyses were performed using R software version 2.72 (http://www.r-project.org/), a commonly used statistical package in the public domain. The analytic result captures the temporal patterns of CPS, CPD, IMR and LOS, which may be directly or indirectly influenced by healthcare reforms. Like any typical observation studies, the current study does not lead to any causal inferences.

## Results

### Study Hospitals and Patients

Table 1 shows the characteristics of three hospitals and hospitalized patients. Total bed numbers of hospital A, B and C were 1,500, 1,381 and 1,190 in 2010, which increased by 9.6%, 13.3% and 0.6%, respectively, from 2006 to 2010. The total numbers of hospital discharges in the three hospitals were, respectively, 45,638, 47,276, and 67,343 in 2010, increasing by 20.2% to 78.7% compared with the year 2006. Most of patients were females (56.7%) and those aged at 30–64 years (51.2%). In addition, out-of-pocket payment accounted for 50.7%, basic social medical insurance (BSMI) 23.2%, and governmental insurance 18.6%, while commercial insurance accounted for only 0.1%.

**Table 1:** Descriptions of the Study Hospitals and Inpatients.

	Hospital A		Hospital B		Hospital C		Total	
*Hospital characteristics*								
Number of beds								
Year 2006	1368		1219		1183		3770	
Year 2010	1500		1381		1190		4071	
Change	+9.6%		+13.3%		+0.6%		+8.0%	
Total hospitalization								
Year 2006	37972		33577		37690		109239	
Year 2010	45638		47276		67343		160257	
Change	+20.2%		+40.8%		+78.7%		+46.7%	
*Inpatient characteristics*	No.	(%)	No.	(%)	No.	(%)	No.	(%)
Total hospitalization	206294	(100.0)	200800	(100.0)	244465	(100.0)	651559	(100.0)
Gender								
Female	114874	(55.7)	111603	(55.6)	142831	(58.4)	369308	(56.7)
Male	91420	(44.3)	89197	(44.4)	101634	(41.6)	282251	(43.3)
Age (years)								
0–1	11521	(5.6)	3182	(1.6)	6832	(2.8)	21535	(3.3)
2–6	8916	(4.3)	3965	(2.0)	3491	(1.4)	16372	(2.5)
7–18	15605	(7.6)	10847	(5.4)	6880	(2.8)	33332	(5.1)
19–29	22852	(11.1)	24947	(12.4)	42458	(17.4)	90257	(13.9)
30–49	53645	(26.0)	54798	(27.3)	86575	(35.4)	195018	(29.9)
50–64	44251	(21.5)	49100	(24.5)	45689	(18.7)	139040	(21.3)
65–74	28926	(14.0)	31510	(15.7)	31907	(13.1)	92343	(14.2)
≥75	20578	(10.0)	22451	(11.2)	20633	(8.4)	63662	(9.8)
Severity at Admission								
Critical	122	(0.1)	122	(0.1)	1099	(0.4)	1343	(0.2)
Severe	33599	(16.3)	13844	(6.9)	14040	(5.7)	61483	(9.4)
General	172573	(83.7)	186834	(93.0)	229326	(93.8)	588733	(90.4)
Type of payment								
BSMI†	66163	(32.1)	11096	(5.5)	73960	(30.3)	151219	(23.2)
Commercial	84	(0.0)	6	(0.0)	490	(0.2)	580	(0.1)
Out-of-pocket	66698	(32.3)	130581	(65.0)	133186	(54.5)	330465	(50.7)
Governmental	62821	(30.5)	21972	(10.9)	36630	(15.0)	121423	(18.6)
CASD‡	23	(0.0)	5503	(2.7)	5	(0.0)	5531	(0.8)
Other	10505	(5.1)	31642	(15.8)	194	(0.1)	42341	(6.5)

† Basic social medical insurance.

‡ Comprehensive arrangement for serious disease.

### Hospitalization Costs

**Figure 1:**
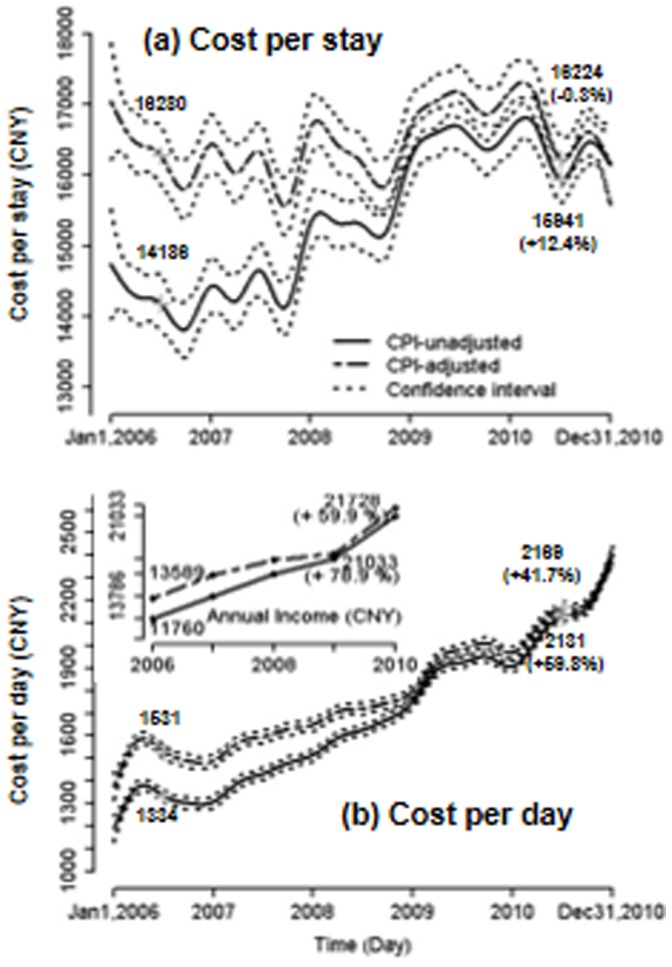
(a) Cost per stay, and (b) Cost per Day between 2006 and 2010 in the 3 teaching hospital. All costs displayed are for an individual hospitalization. For comparison, national annual income per head is embedded in Figure 1b.

Figure 1a shows the CPI-adjusted and CPI-unadjusted temporal trends of CPS in three hospitals. As expected, the CPI-adjusted CPS was relatively higher than the CPI-unadjusted CPS, the latter of which is often mentioned in public media. While unadjusted cost was steadily increasing, adjusted cost was actually stable during 2006 to 2010. In contrast, CPD, with and without CPI adjustment, were persistently increasing by 41.7% and 59.8%, respectively (Figure 1b). The CPI-adjusted CPD increased from CNY 1,531 in 2006 to CNY 2,169 in 2010. During this period of time, the average CPI-adjusted national annual income per capita in China was also steadily increasing by 60%, from CNY 13,589 to CNY 21,728, which is about 12% every year (the embedded figure in Figure 1b).

**Figure 2:**
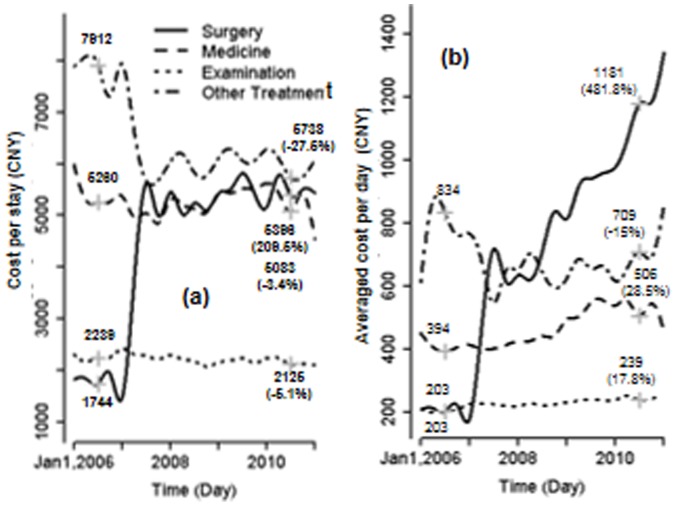
(a) Costs per stay by surgery, prescription drug, examination and other treatments and (b) averaged costs per day by the four categories. All costs displayed are for an individual hospitalization.

Figure 2a shows CPS breakdown by four major categories: surgery, medicines, examination, and other treatment. Total surgery cost per stay saw a large jump in early 2007 after a slight decline, and quickly stabilized by the end of year 2010. Overall, total surgery cost increased by about 210% between 2006 and 2010, while other total costs including medicine and clinical examination costs slightly decreased over time. In contrast, surgery cost per day significantly increased by 482% over the 5 years, and examination and medicine costs per day increased by 18% and 29%, respectively (Figure 2b). The temporal trends identified by the adjusted analysis of hospitals, age and gender were similar (not shown as figure). The large jump in the surgery cost in early 2007 was associated with the re-classification of various costs (e.g., devices, suppliers, procedures) at that time, and is presented here to retain the integrity of reported HSRs.

### Health-care Quality and Length of Stay

To explore whether or not quality of care has changed over time, we analyzed the temporal trends of IMR and LOS (data were not shown as figure). Overall, IMR from all causes presented a persistently downward trend, despite the fact that there was a fluctuation, in which IMR fell 36.3% from 1.13% in 2006 to 0.72% in 2010 (P<0.001). In contrast, average length of stay also decreased by 24.8% over time, from 12.1 days in 2006 to 9.1 days in 2010.

### Inpatient Costs and Care by Hospitals

**Figure 3:**
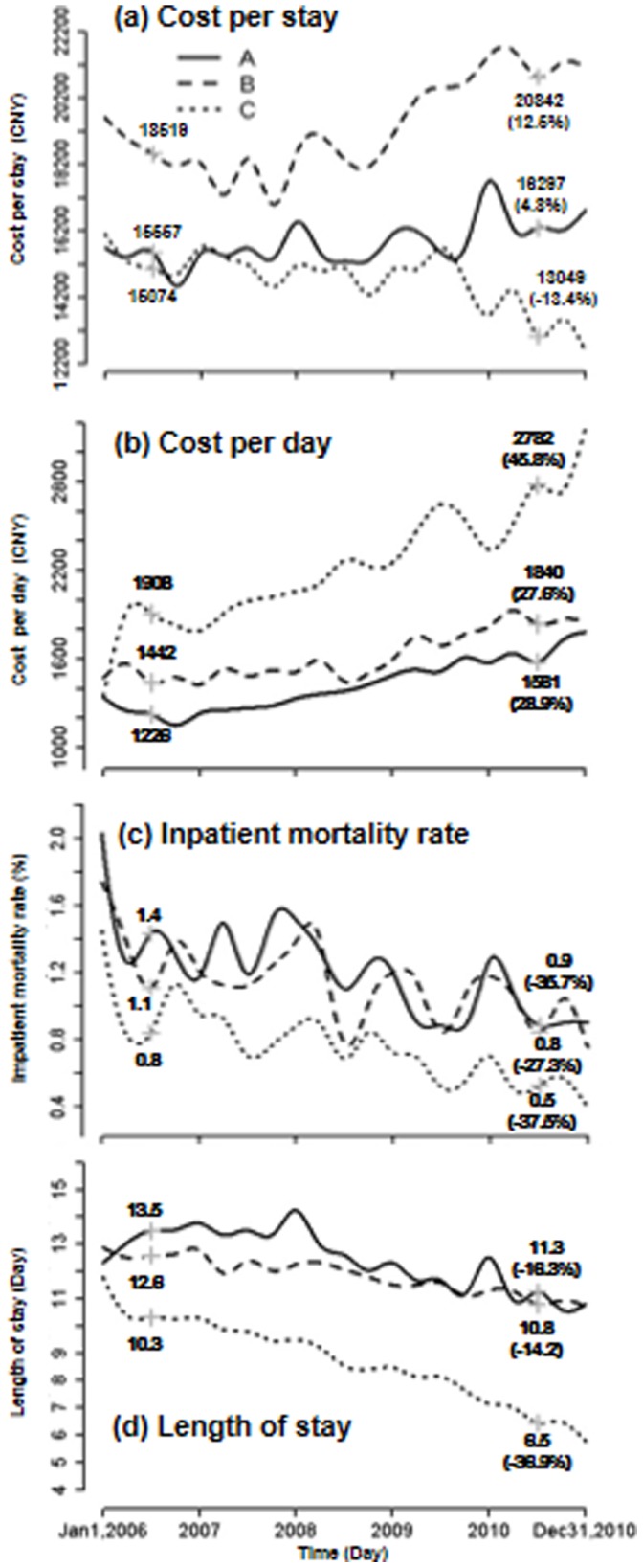
Costs per stay, costs per day, inpatient mortality rates, and lengths of stay between 2006 and 2010 in the 3 teaching hospitals.

Figure 3 displays the temporal trends of CPS, CPD, IMR and LOS by the three hospitals. In 2010, CPS was highest at hospital B (CNY 20,842), moderate at hospital A (CNY 16,297), and lowest at hospital C (CNY 13,049). In terms of CPS, both hospital A and B increased respectively by 4.8% and 12.5% between 2006 and 2010, while hospital C reduced by 13.4%. In contrast, CPD presented a reverse trend pattern, in which hospital C substantially increased CPD by 45.8%, which was much greater than those of hospital A (28.9%) and B (27.6%). Meanwhile, hospital C had a larger decrease in IMR (−37.5%) and LOS (−36.9%). In contrast, hospital A and B with mild increases in CPD both showed relatively moderate decreases in IMR (−35.7% and −27.3%), and particularly in LOS (−16.3% and −14.2%).

### Components in the Cost Growth by 20 Major Hospitalizations

To explore the source of the growth in CPD, we calculated hospital daily revenue ( = CPD×number of admissions per day) for each hospitalization cause in 2006 and in 2010, and then the percent changes of hospital daily revenue between these two years. By the magnitude of percent change, we determined the top 20 major causes for hospitalizations with largest revenue increases. As expected, these 20 hospitalizations were major contributors for the increased CPD.

**Figure 4:**
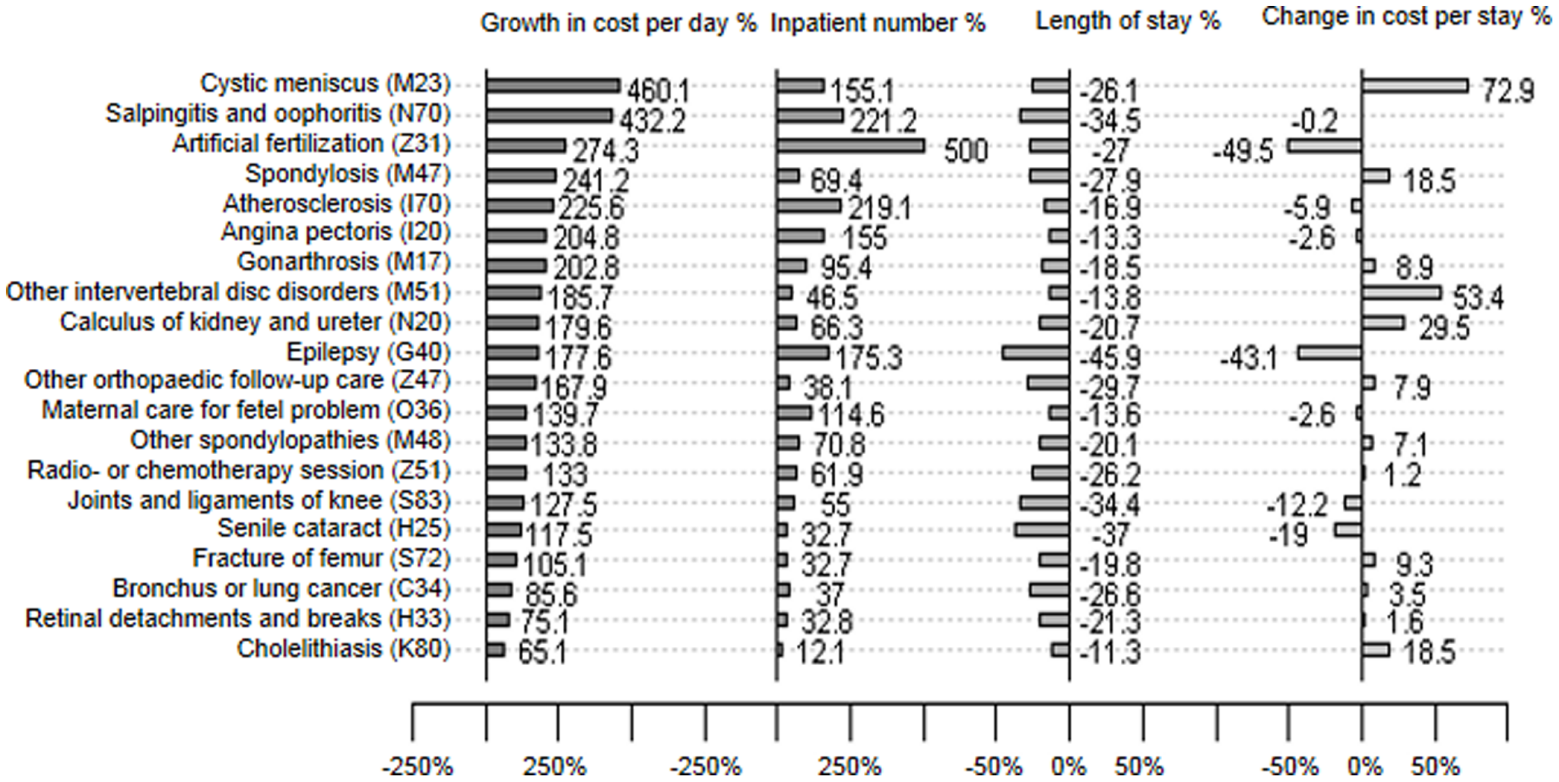
Average change rates (%) between 2006 and 2010 in cost per day, inpatient number, length of stay, and cost per stay among the 20 mostly increased reasons for a hospital stay in the 3 teaching hospitals. The three characters in bracket on the right of hospitalization names are the preceding ICD-10 codes.

Figure 4 shows the 5-year average change rates between 2006 and 2010 in CPD, hospitalized patient number, LOS, and CPS for these 20 causes of hospitalizations. CPD of these hospitalizations grew 65% or more. Six grew even more than 200%. They were cystic meniscus (460%), salpingitis and oophoritis (432%), artificial fertilization (274%), spondylosis (241%), atherosclerosis (225%), and agina pectoris (205%). The numbers of patients within these 20 hospitalizations significantly increased during the 5 years, ranging from 12% (cholelithiasis) to 500% (artificial fertilization). Meanwhile, LOS fell significantly at a range from 11% (cholelithiasis) to 46% (epilepsy), making CPS to increase slowly or even to decrease over time.

## Discussion

In the analysis of 651,559 hospitalizations in three general teaching hospitals in China, inflation-adjusted CPS were relatively stable between 2006 and 2010, but CPD increased at a rate of 8% per year, resulting in a diverging temporal trend pattern. Surprisingly, both IMR and LOS significantly decreased over time. This large analysis not only provides reliable estimates of inpatient cost, but also shows the sound deliveries of cost-controlled and quality services in these three teaching hospitals, and offers an opportunity using the first-hand data to depict how these hospitals are adapting to the market-oriented health-care reform in China.

Hospitalization costs represent the largest component of health care expenditures in most hospitals. As a result, controlling its growth is of primary concern to Chinese government, and also directly associated with the evaluation of the health-care reforms. By analyzing these three teaching hospitals, we noted that CPS had been adequately controlled from year 2006 to 2010, in compliance with the governmental regulations. Moreover, the proportion of a single CPS to national annual income per capita had actually been reduced to 75% in 2010 from 120% in 2006. The current proportion of 75% in 2010 in these three teaching hospitals was about 15% lower than the average national level of all hospitals reported in 2006 [3]. While cheering for this meaningful control of CPS, we need to realize that much of the cost control is due to shortening LOS, despite the growth of CPD. Indeed, average LOS was about 30% shorter in 2010 than in 2006. Figure 4 showed how CPS was reduced with the decreasing LOS. The findings uncovered the key strategy that hospitals attempted during the health-care reform, that is to improve care quality and keep up with the economic development, while controlling CPS through shortening LOS and increasing hospitalizations with higher profit margins.

The growth of CPD in these three hospitals was proportionally synchronous with the growth of national annual income per capita. Average CPD was CNY 2,169 (∼US $339) in 2010, which was more than 6-fold lower than the CPD of US $2,000 in the U.S [6]. While the CPD rose persistently over time, its growth slowed down to only 8% per year, as compared with the large jump of more than tenfold between 1980 and 2000, indicating that the excessive growth of inpatient cost has been controlled to certain extent.

As expected, to keep up with the economic development, hospitals were motivated to maximize the hospitalization revenues. Significantly increased high-revenue hospitalizations (Figure 4) demonstrated the fact that a hospital was motivated to provide more profitable services than others [7]. While the revenue-driven hospitalizations in these three hospitals seemed rational under the current market-oriented health-care reform, this trend is probably not sustainable and, probably, is not necessarily desirable in the context of overall public health.

Multiple factors probably contribute to declining IMRs in these three hospitals. One of the most important factors is the increased use of modern medical technologies such as new medicines, new antibiotics, and minimally invasive surgical techniques. Although cost is somewhat high, these new medical technologies substantially reduce the chances of surgical infections and complications, and ultimately reduce the IMR. Another responsible factors are the improved facilities for clinical care or improved hospital managements in hospitals. These three hospitals we analyzed had invested substantial portions of their revenues on improving hospital facilities and capabilities. Many previous studies have found that the increase of hospital spending was associated with higher quality care, particularly in terms of reducing IMR. Lastly, with the improving personal incomes and the increasing health insurance coverage, patients are more likely to seek health-care earlier, before the disease conditions became too severe. Using a subjectively judged disease severity at admission, reported on HSR, we noted that the overall improvement of disease severity at admission from year 2006 to 2010 (not shown).

There are opportunities to further control hospitalization costs through shortening LOS in the three hospitals. As noted above, the LOS in these three hospitals has been steadily declining. As of 2010, the average LOS in these hospitals is still around 9.1 days, which is nearly twice as high as the average of 4.6 days in the United States [6]. In fact, some treatments could be performed without hospitalizations. For example, cataract patients are hospitalized for a surgical treatment in China, while such surgery is generally performed at ambulatory services in the U.S. Nevertheless, it is important to note that excessive reduction in LOS is not warranted without harming patients’ interest. Also, a successful reduction of LOS also requires the support from other health-care providers who can continue nursing-equivalent cares after leaving hospitals.

We noted that the costs of medicines and examinations had been relatively stable in these three hospitals. Between 2006 and 2010, their costs per day increased only by about 28.5% and 17.8%, respectively. In contrast, surgery cost per day increased by about 64% ((1184–720)/720×100). This growth rate was calculated by removing the effect of a large jump due to cost re-classification in early 2007. The increase in surgery cost was highly correlated with the uses of new surgical technologies. We also noted that the number of hospitalization increased by 47% between 2006 and 2010 in the three hospitals (Table 1). The increase of admissions also reflected the hospital efficiency.

While appreciating all of these changes during this critical period, it is important to realize that no reform plan was designed to target at public hospitals, such as service quality, patient safety and financial interests. What we have observed is reflective of how these three teaching hospitals were adapting to the changing environment. Through shortening LOS, hospitals are able to increase productivities for controlling costs. As far as increasing CPD, it is proportionally synchronous with the increase of national annual income per capita. Equally importantly, IMR was steadily declining, implying improving patient safety and service quality.

One critical issue, underlying all of results presented above, is the data quality of the HSR data, which has been a common criticism to data collected from large population studies in China. However, the HSR data, used in this report, may be one of exceptions. First of all, all three hospitals studied here are general teaching hospitals, and enjoy the prestigious esteems for their quality in all aspects of health-care, including electronic medical record systems. Secondly, HSR data were required by the Beijing Municipal Health Bureau for administrative purposes, including the evaluation of hospital performance, the allocation of financial resources, and the development of Disease Related Groups (DRG). Thirdly, because of the administrative requirement, each hospital is asked to send in HSR on a fixed schedule (weekly or monthly), with minimum opportunities to alter reported data. Lastly, the Beijing Municipal Health Bureau had recently conducted quality control study, and found that over 95% of hospitalization diagnostic codes on the HSR data were accurate, based upon manual examinations of electronic medical records (personnel communication with the Bureau).

There are a few limitations worthy commenting. Firstly, three Peking University hospitals are attracting not only local patients but also those from other regions in China. While the referral bias in hospital-based studies is often of concern, results obtained in this study are likely to represent those similar top hospitals in Beijing and also in other large cities of China. Since these three hospitals are not randomly chosen, results obtained here are best confined to these hospitals, which are a tip of iceberg for a wide range of public hospitals in China. Secondly, the classification of hospitalization costs has undergone changes from time to time, e.g., the cost-breakdown of surgery cost has been re-classified, resulting in a jump around 2007. We have chosen not to make any adjustment to this jump, to keep the integrity of cost data. Further, reported CPS and CPD are reliable, and are consistently collected during this period. Thirdly, the HSR is an abstract of hospitalization, lacking clinical details that are necessary to produce other important clinical indicators, such as in-hospital infections and clinical pathways. As electronic medical records are more widely adopted, however, it may be possible to extract more complete HSR, producing comprehensive evaluation indicators in the future. Fourthly, it is important to recognize that this study is observational, and results from analyses are restricted to be temporal changes of multiple factors. Hence, results should not be interpreted as causal inferences, since many other factors, together with healthcare reform, could contribute to these temporal patterns observed here. Lastly, but not least, the HSR data lack reporting on patients’ satisfaction, and hence prohibit a direct assessment from the patient’s perspective.

The temporal trends, documented in this manuscript, reflect how hospitals are evolving throughout the health-care reforms. Earlier, health-care was financially supported entirely by Chinese government. Since the mid-1980s, the government began to reduce its budget as percentages of total hospital revenues. By the end of 2000s, the budget accounted for less than 10% percent of typical public hospital revenue, while the remainders were from medical service fees and drug sales [8]. Since 2009, government has successfully launched new insurance program, hence covering nearly 95% of the population. This reform policy would certainly increase accesses to hospitals. Currently in year 2012, government is now ready to reform public hospitals, controlling excessive prescriptions by eliminating added fees to drug prescriptions. As expected, such reform policies will substantially impact the health-care delivery efficiency, incentives and qualities, some of which are implied by this study.

Unlike western countries, China focuses its reform on patients’ needs and interests thus far, and now shifts towards reforming public hospitals. During this period of time, many hospitals have been adapting, and as we found some top hospitals were emerging with some promising characteristics: high efficiency, improved safety and high revenue. While there are still many unresolved problems, the current results of these three teaching hospitals by the self-governances under government regulation and surveillance may be used as reference not only for future health-care reform in China, but also for other emerging countries with less government supervision for public hospitals, such as India.^4^ On the other hand, health-care has been an international concern, from developing countries to developed countries. Understanding the health-care reform in China, a diverse country from poor to wealthy cities/provinces, would also shed light on consequences of health-care reform on health-care delivery system. In fact, controlling cost and enhancing care quality are always the primary purpose concerned by various health-care delivery systems.

### Conclusions

In conclusion, this temporal analysis of costs, inpatient mortality and length of stay provides the first-hand data on how top rank public hospitals are adapting to the changing environment during the health-care reform during the period from 2006 to 2010. Our findings have an implication to future reforms of large public hospitals. In the absence of major health-care reform targeted at public hospitals, large public hospitals had already taken an initiative of adapting to the changing environment. Many changes are positive; controlling cost, improving quality, and growing services. To sustain these positive changes towards modern health-care systems, the next phase of the health-care reform should be designed to incentivize hospitals at various levels to perform according to expectations, to strengthen financial support towards infrastructures, to delink personal incentives with health-care services, and to improve recognitions for health-care providers in society.

As China continues its health-care reform in next few years, the world is watching the largest “social experiment” with probably the most populous country on this planet. Lessons from this experiment will have a lot to offer to other developing and developed countries, as the health-care cost becomes increasingly concerns in the world. For accurate assessments of costs, quality and coverage, it is important for an independent organization to continuously monitor the progresses of the health-care reform, from perspectives of both health-care providers and general public, using actual health-care data directly obtained from hospitals and from patients.
